# Efficacy of diode and CO_2_ lasers along with calcium and fluoride-containing compounds for the remineralization of primary teeth

**DOI:** 10.1186/s12903-019-0813-6

**Published:** 2019-06-19

**Authors:** Elham Soltanimehr, Ehsan Bahrampour, Zeynab Yousefvand

**Affiliations:** 1 Department of Pediatric Dentistry, School of Dentistry, Kermanshah University of Medical Sciences, Kermanshah, Iran; 2 Department of Oral and Maxillofacial Radiology, School of Dentistry, Kermanshah University of Medical Sciences, Building No. 1, Shahid Beheshti Boulevard, Kermanshah, 6715847141 Iran; 3 School of Dentistry, Kermanshah University of Medical Sciences, Kermanshah, Iran

**Keywords:** Diode laser, CO_2_ laser, Remineralization, Casein Phosphopeptide amorphous calcium phosphate, Tricalcium phosphate fluoride, Microhardness, Porosity

## Abstract

**Background:**

This study aimed to assess the efficacy of a 980-nm diode and 10.6-μm CO_2_ laser accompanied by tricalcium phosphate-5% sodium fluoride (fTCP) and casein phosphopeptide amorphous calcium phosphate (CPP-ACP) for the remineralization of primary teeth.

**Methods:**

In total, 117 extracted primary anterior teeth were randomly divided into eight experimental and one control group: (I) control (polished enamel), (II) fTCP varnish, (III) fTCP + diode laser, (IV) fTCP + CO_2_ laser, (V) CPP-ACP, (VI) CPP-ACP + diode laser, (VII) CPP-ACP + CO_2_ laser, (VIII) diode laser, and (IX) CO_2_ laser. The microhardness of 12 samples in each group and the enamel porosity of one sample in each group were assessed before and after demineralization and 28 days after remineralization. Data were analysed using two-way ANOVA.

**Results:**

Significant differences existed in microhardness (*P* = 0.004) and percentage of remineralization (*P* < 0.001) after remineralization among the material groups such that the highest mean was noted in the CPP-ACP group. No significant difference was noted in microhardness (*P* = 0.052) or percentage of remineralization (*P* = 0.981) after remineralization among the laser groups. In all groups, porosities increased after demineralization and slightly decreased after remineralization; the greatest reduction in porosity of the material groups was noted in the fTCP group, and the CO_2_ group among the laser groups. The interaction effect of materials and lasers was not significant (*P* > 0.05).

**Conclusion:**

The highest microhardness was achieved after remineralization with CPP-ACP. The efficacy of the diode and CO_2_ lasers was the same. No synergistic effect was found between materials and lasers.

**Trial registration:**

This is not a human subject research.

## Background

Dental caries in primary teeth is a common problem in most developed and developing countries [1]. Tooth decay occurs as the result of an imbalance between demineralization and remineralization [2]. To maintain this balance, an adequate amount of calcium, phosphate and fluoride ions must be present in the oral environment [2]. Thus, several non-invasive methods have been suggested to provide these ions in the oral cavity [3, 4]. Calcium and phosphate ions form the main structural component of the enamel, i.e., hydroxyapatite [5]. An increase in the concentration of these ions in the oral cavity and dental plaque can cause enamel remineralization [2]. Several materials have been introduced to increase the concentration of these ions in dental plaque. Casein phosphopeptide amorphous calcium phosphate (CPP-ACP) is among the suggested materials for this purpose, which is composed of two parts of CPP and ACP; CPP is a nanocomplex derived from milk protein (casein). It can stabilize calcium and phosphate in a solution and increase their concentration [6]. CPP-ACP is used for the remineralization of incipient caries in primary teeth, and it has been noted that it remineralizes enamel and increases the size of hydroxyapatite crystals [7]. Tricalcium phosphate (TCP) varnish has also been suggested for caries prevention and enhancing remineralization, which has an alcohol base [3]. Functionalized TCP (fTCP) is prepared by combining TCP and sodium lauryl sulphate or fumaric acid. Clinpro White varnish is a fTCP varnish containing 5% sodium fluoride, calcium and phosphate. Its advantages include easy use, no need for tooth isolation and the ability to bond to dry or wet teeth. It also occludes the dentinal tubules and decreases tooth hypersensitivity [8]. It prevents dental caries and increases surface microhardness because it contains ions necessary for remineralization [9].

Evidence shows that laser irradiation can change enamel composition and increase its resistance to acid dissolution and caries [10, 11]. Aside from chemical and structural changes [12], laser irradiation increases the uptake of fluoride ions and remineralization and decreases caries as such [13]. Several laser types have been used for this purpose [11–20], but there is still controversy regarding their efficacy [16–20]. Most previous studies have used CO_2_ lasers for this purpose [11–18], but studies on diode lasers are limited [19, 20]. Seino et al. showed that CO2 laser alone was able to control enamel demineralization at the same level as that obtained with topical fluoride application [16]. Souza-Gabriel et al. stated that CO2 laser association with concentrated fluoride therapy may not result in a positive synergistic interaction to decrease the demineralization of subsurface enamel [17] while Zancopé et al. and González-Rodríguez et al. demonstrated that laser irradiation of the enamel surface can increase fluoride uptake [18, 20].

Therefore, this study aimed to compare the efficacy of CO_2_ and diode laser irradiation accompanied by the use of compounds containing calcium and fluoride for remineralization of primary teeth.

## Methods

This in vitro experimental study was conducted on extracted primary anterior teeth of 5–7-year-olds presenting to two dental clinics in Kermanshah. All parents provided their informed consent in writing. The teeth were selected using convenience sampling. The sample size was calculated to be 13 in each group according to a previous study by Memarpour et al., (2015). The study was approved by the ethics committee of our university.

A total of 117 extracted human sound primary anterior teeth were disinfected in 0.1% chloramine T solution and stored in distilled water at 37 °C. The roots were cut 2 mm below the cementoenamel junction. The crowns were evaluated under a stereomicroscope (Motic, Wetzlar, Germany) at × 40 magnification to exclude teeth with defects, cracks, caries, wear or stains. The teeth were then mounted in an epoxy resin mould (Struers A/S, Ballerup, Denmark) such that their surface was at the level of the mould surface and they were located at the centre of the mould. To obtain a smooth surface, enamel was ground using 600-, 800- and 2400-grit silicon carbide and 1-, 2- and 3-μm aluminium oxide papers followed by 30 s of rinsing with distilled water and drying. The teeth were then coded and randomly divided into eight experimental groups and one control group (*n* = 13). The enamel microhardness test was performed for 12 samples in each group before and after demineralization and 28 days after remineralization. One sample of each group was also used for atomic force microscopy (AFM). These teeth were not used in microhardness tests.

### Enamel microhardness test

A sticker was placed on the buccal surface of the teeth measuring 2 × 2 mm, and its surrounding area was sealed with two layers of nail varnish. The sticker was then removed, and excess material was rinsed with distilled water. This was done to standardize the tooth surface subjected to materials. The microhardness of the tooth surface was measured by applying a 50-g load for 10 s to five points of each sample using a Vickers diamond indenter (Wolpert, Darmstadt, Germany). The Vickers hardness number (VHN) was determined by calculating the ratio of load (kilogram force) to the surface area (mm^2^) using the formula below [9]:

$$ \mathrm{VHN}=\mathrm{F}/\mathrm{A}=1.8544\mathrm{F}/\mathrm{d}2 $$

Where F is the applied load in kilogram force and A is the surface area in square millimetres.

After obtaining the VHN, the mean microhardness of the five points was calculated and reported as the microhardness of enamel. All teeth were then demineralized to create incipient caries.

### Demineralizing solution

Each sample was placed in a separate container containing 50 mL of demineralizing solution at 37 °C for 96 h. This solution contained 0.1 mM lactic acid, 3 mM calcium chloride, 3 mM potassium dihydrogen phosphate and 0.2 guar gum [21]. The final pH was adjusted to 4.5 using 50% sodium hydroxide [22]. This solution was refreshed after 48 h. After 96 h, each sample was rinsed with deionized water for 20 s and air-dried for the second microhardness test of demineralized enamel.

### Test groups

After the second measurement of microhardness, the following materials were used for enamel surface remineralization. Table 1 shows the materials used in this study and their composition.

**Table 1:** Materials used in this study and their composition

Material	Composition	Manufacturer
Tooth Mousse (Recaldent)	Pure water, Glycerol, CPP-ACP, D-sorbitol, CMC-Na, Propylene glycol, Silicone dioxide, Titanium dioxide, Xylitol, Phosphoric acid, Zinc oxide, Sodium Saccharin, Ethyl p-hydroxybenzoate, Magnesium oxide, Guar gum, Propyl p-hydroxybenzoate, Butyl p- hydroxybenzoate	GC, Tokyo, Japan
Clinpro White Varnish	Tri-Calcium-Phosphate (TCP), Alcohol, Rosin, Sodium fluoride, Xylitol, Sodium Fluoride	3 M, Hackensack, NJ, USA

Group 1. fTCP varnish: One layer of Clinpro White varnish (3 M, Hackensack, NJ, USA) was applied on the tooth surface according to the manufacturer’s instructions. This varnish included TCP and 5% sodium fluoride and was used on 1st and 16th days after rinsing the enamel with deionized water.

Group 2. fTCP + diode laser: Clinpro varnish was applied according to the manufacturer’s instructions followed by 980 nm diode laser irradiation (ARC laser, Fox, Germany) with 7 W power for 15 s.

Group 3. fTCP + CO_2_ laser: Clinpro white varnish was applied according to the manufacturer’s instructions followed by 10.6 μm CO_2_ laser irradiation (Lasersat 15tm, Satelec, Merignac, France) with 2 W power for 15 s.

Group 4. CPP-ACP: One layer of Recaldent (GC, Tokyo, Japan) was applied for four minutes after drying the tooth surface. This crème included 10% CPP-ACP, which was applied twice a day at 8.00 a.m. and 16.00 p.m. for 28 days.

Group 5. CPP-ACP + diode laser: After applying Recaldent, the diode laser was irradiated as explained for group 2.

Group 6. CPP-ACP + CO_2_ laser: Recaldent was applied as in group 4 followed by CO_2_ laser irradiation as in group 3.

Group 7. Diode laser with 980 nm wavelength was irradiated with 7 W power for 15 s.

Group 8. CO_2_ laser with 10.6 μm wavelength and 2 W power was irradiated for 15 s.

Group 9. This group included 13 samples and served as the control group. No intervention was performed for samples in this group. Teeth with demineralized enamel were immersed in distilled water for 28 days and the water was refreshed every 3 days.

After 28 days, the teeth were rinsed with deionized water for 20 s and the enamel microhardness of each sample was measured. The percentage of recovery of enamel microhardness (%REMH) was calculated as follows [9]:

$$ \%\mathrm{REMH}=\left[\mathrm{Microhardness}\ \mathrm{of}\ \mathrm{remineralized}\ \mathrm{enamel}-\mathrm{microhardness}\ \mathrm{of}\ \mathrm{demineralized}\ \mathrm{enamel}\right]\times 100/\left[\mathrm{microhardness}\ \mathrm{of}\ \mathrm{sound}\ \mathrm{tooth}-\mathrm{microhardness}\ \mathrm{of}\ \mathrm{demineralized}\ \mathrm{enamel}\right] $$

Microhardness was measured using a Vickers hardness tester (Wopert, Darmstadt, Germany) with 50 g load for 10 s. The results were reported in Newtons per square meter (N/m^2^).

### Remineralizing solution

The samples were immersed in a remineralizing solution for 28 days, which included 2.200 g/L gastric mucin, 0.381 g/L sodium chloride, 0.213 g/L calcium hydrogen chloride, 0.738 g/L potassium hydrogen phosphate and 1.114 g/L potassium chloride [7, 8, 22]. The final pH was adjusted at 7 using 85% lactic acid at 37 °C. This solution was refreshed every 48 h.

### Atomic force microscopy

The topographic characteristics of the enamel surface were assessed using AFM. One sample of each group was used for AFM (JPK Nanowizard II apparatus, JPK Instruments, Berlin, Germany) in tapping mode along with a nonconductive silicon nitrite cantilever (Acta-Probe, APPNano, CA, USA) and a piezoelectric scanner. Scanning frequency was 1 Hz and the spring constant was 13 N/m. The mean surface roughness was measured in five areas (each measuring 5 × 5 μm) using the formula below [23]. The results were recorded in nanometres (nm).

$$ \mathrm{Ra}=\frac{1}{N}{\sum}_{i=1}^N\left|{Z}_i-\overline{Z}\right| $$

Where N is the number of points assessed and Zi-Z is the height relative to the middle surface [23]. The mean hardness was calculated for each sample.

### Statistical analysis

Data were analysed using SPSS version 17 (SPSS Inc., Chicago, IL, USA). The Kolmogorov-Smirnov test was used to assess the normal distribution of data. Considering the normal distribution of data (*P* > 0.05), the enamel microhardness value after remineralization and the change in enamel microhardness after remineralization (%REMH) were compared among the groups using the two-way ANOVA. The Tukey’s post hoc HSD test was used for pairwise comparisons. The significance level was set at 0.05.

## Results

In this study, 108 teeth were subjected to microhardness test and nine teeth were subjected to AFM. A total of 117 teeth were randomly divided into eight experimental groups and one control group. During the experiment and after the statistical analysis, we noticed that the findings related to one tooth were unexpectedly different from the values obtained for other teeth. After further scrutiny, we found that the respective tooth was carious and its carious lesion had been somehow missed in the initial screening process for inclusion of samples.

**Fig. 1:**
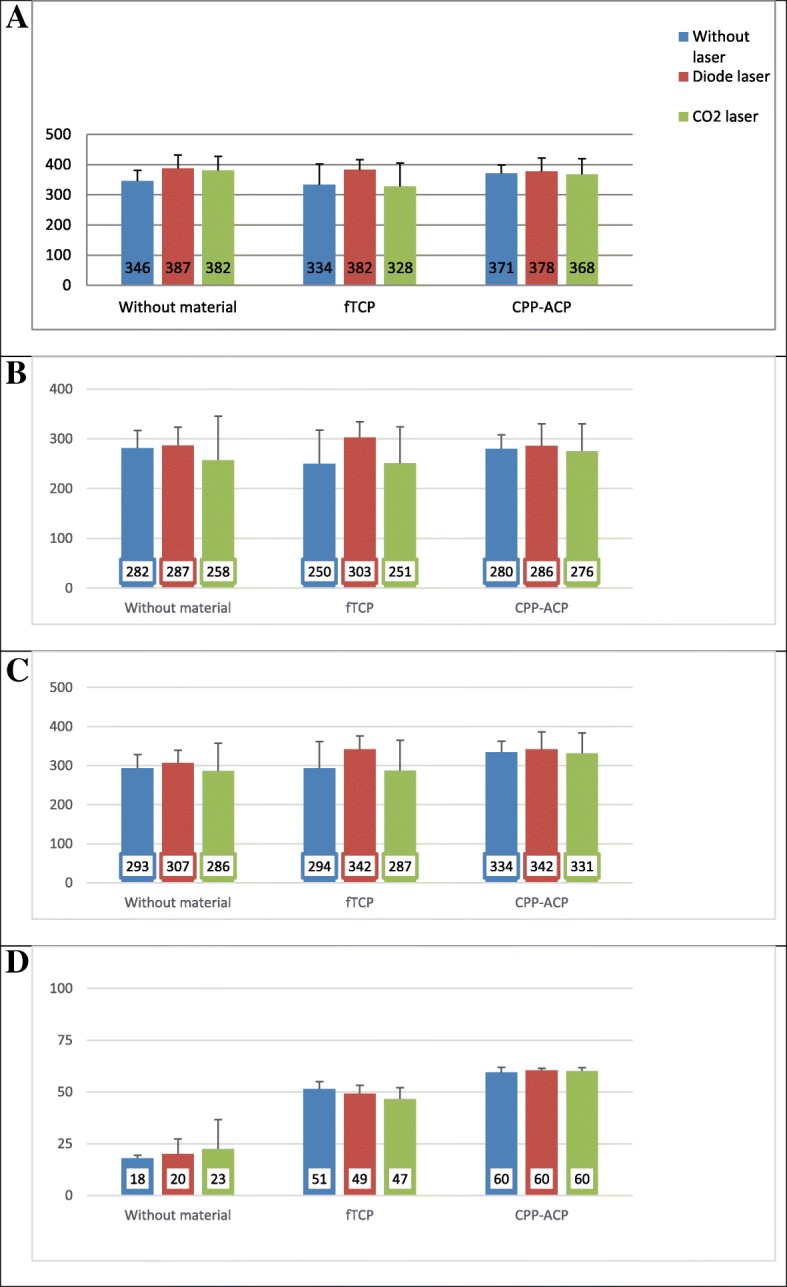
**a** Primary microhardness of samples according to the type of laser and material used. **b** Mean microhardness after demineralization according to the type of laser and type of material. **c** Microhardness after remineralization according to the type of laser and material. **d** Percentage of remineralization according to the type of laser and material used

Figure 1(a) shows the primary microhardness of samples according to the type of laser and material used. Two-way ANOVA showed a significant difference in primary microhardness of the laser groups (*P* = 0.021). The lowest and the highest mean values belonged to the no laser and diode laser groups, respectively. No significant difference was noted in the primary microhardness of the material groups (*P* = 0.071). Figure 1(b) shows the mean microhardness after demineralization according to the type of laser and type of material used. Two-way ANOVA showed no significant difference in microhardness after demineralization among the laser groups (*P* = 0.062). No significant difference was noted in microhardness of material groups after demineralization (*P* = 0.633). Figure 1(c) shows the microhardness after remineralization according to the type of laser and material used. Two-way ANOVA showed no significant difference in microhardness after remineralization among the laser groups (*P* = 0.052). A significant difference existed in microhardness after remineralization among material groups (*P* = 0.004) such that the lowest mean value was noted in the no-material group and the highest mean value was noted in the CPP-ACP group. Figure 1(d) shows the mean percentage of remineralization according to the type of laser and material used. Two-way ANOVA found no significant difference in the percentage of remineralization among the laser groups (*P* = 0.981). A significant difference was noted in the percentage of remineralization among the material groups (*P* < 0.001) such that the lowest mean value was noted in the no-material group and the highest mean was noted in the CPP-ACP group.

**Fig. 2:**
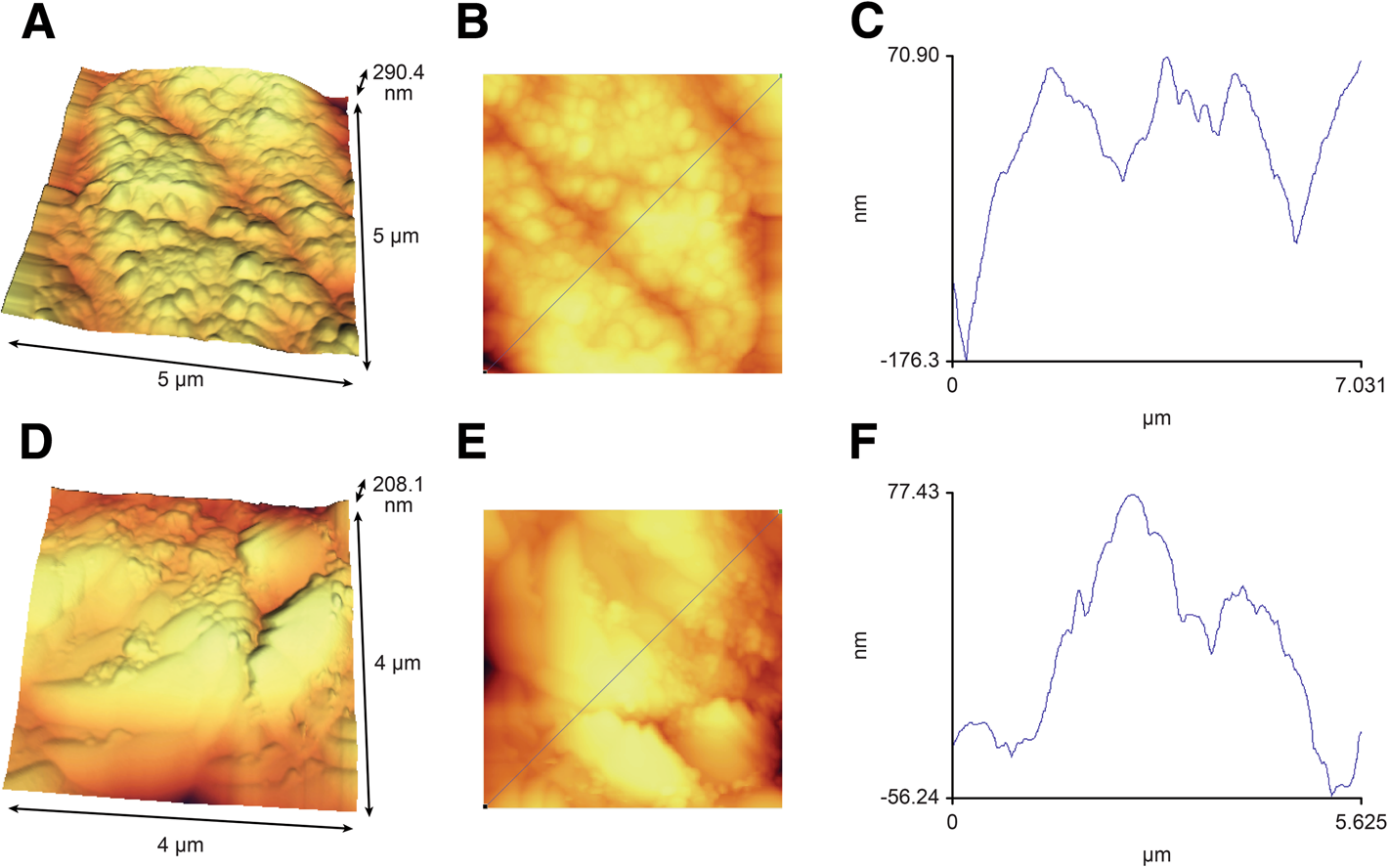
**a**–**c** Enamel demineralization (5 × 5 μm); mean porosity: 111.65 nm; distance between the highest and lowest point: 290.4 nm (**d**–**f**) 5 × 5 μm area treated with fTCP and diode laser; mean porosity: 96.74 nm; distance between the highest and lowest point: 208.1 nm

Table 2 shows the mean and standard deviation of porosity in the groups. In all material groups, the porosity rate increased after demineralization but slightly decreased after remineralization. The greatest reduction in porosity after remineralization was noted in the fTCP group. In all laser groups, the porosity rate increased after demineralization but slightly decreased after remineralization. The greatest reduction in porosity after remineralization was noted in the CO_2_ group. Figure 2 shows the AFM images of the surface of samples.

**Table 2:** Mean and standard deviation of porosity in the groups

Group	AFM1		AFM2		AFM3	
	Mean	Standard deviation	Mean	Standard deviation	Mean	Standard deviation
No material	13.06	.21	115.48	5.88	101.90	3.24
fTCP	13.33	1.54	118.35	3.59	92.74	6.69
CPP-ACP	13.38	.90	121.79	6.38	96.92	1.34
No laser	12.86	.23	121.57	7.42	101.34	3.75
Diode	14.09	1.08	117.69	3.78	96.14	5.35
CO2	12.83	.73	116.36	5.18	94.09	5.97

*AFM1* AFM before demineralization, *AFM2* AFM after demineralization, *AFM3* AFM after remineralization

## Discussion

This study aimed to assess the efficacy of 980-nm diode and 10.6-μm CO_2_ lasers accompanied by fTCP and CPP-ACP for the remineralization of primary teeth. The results showed a significant difference in primary microhardness of the groups but after demineralization, the difference between the laser and material groups was not significant, and the mean microhardness decreased after demineralization. The microhardness values measured in our study were similar to the values reported in previous studies [24, 25]. Slight differences may be attributed to the method of measurement of the microhardness of enamel, the difference in the type of teeth, their composition, age of patients, storage medium and study design.

Silverstone was the first to discuss that remineralization by salivary calcium and phosphorus ions is a slow process, and due to the low concentration gradient, the deposition of minerals mainly occurs in the superficial layer [26]. They added that materials other than saliva are required to provide calcium and phosphate ions for the remineralization of lesions. Thus, solutions and varnishes containing calcium, phosphate and fluoride were introduced to enhance the tooth resistance to caries and increase the percentage of remineralization [27]. Our study compared CPP-ACP and fTCP and showed a significant difference in the microhardness of material groups after remineralization. The highest mean microhardness was noted in the CPP-ACP group. The material groups were also different in the percentage of remineralization, and the highest mean value was noted in the CPP-ACP group. Cochrane et al. reported that CPP releases higher amounts of calcium and fluoride than the TCP varnish [28]. Schemehorn et al. compared two varnishes containing calcium and phosphate and showed that Enamel Pro ACP transferred higher amounts of fluoride to the demineralized enamel compared with TCP [29]. However, the results of some previous studies were different from ours. Rirattanapong et al. found that CPP paste and TCP varnish equally increased the microhardness of permanent teeth [30]. Patil et al. used DIAGNOdent and showed that the effect of TCP was greater than CPP. This difference in the results may be due to the technique used to assess the surface after remineralization, type of tooth, sample size and materials used [22]. Al-Mullahi and Toumba [31] and Yamaguchi et al. [32] stated that CPP-ACP can prevent demineralization and reinforce remineralization, which was in agreement with our findings. CPP bonds to calcium and phosphate via the phosphoserine in its composition and forms small clusters of ACP. Thus, insoluble calcium and phosphate become soluble in the presence of CPP [33]. Additionally, CPP bonds to the tooth surface and serves as a source of calcium and phosphate ions [34]. Calcium and phosphate ions penetrate into the porous structure of lesions and deposit on relatively demineralized apatite crystals, reforming them and enhancing remineralization [35]. Zhou et al. reported that in demineralized enamel, the central parts of the prisms are replaced with CPP-ACP in the process of remineralization over time, and crystals maintain their orientation while the enamel surface gradually becomes smoother [7]. These results were confirmed with the surface microhardness findings. Zhou et al. indicated that CPP-ACP and NaF can decrease the surface roughness of incipient enamel lesions in primary teeth by remineralization and increase their hardness and modulus of elasticity [7]. In the current study, the rate of porosity increased in material groups after demineralization and slightly decreased after remineralization. The greatest reduction in porosity after remineralization was noted in the fTCP group. To prevent unfavourable interactions of calcium, phosphate and fluoride, an acidic barrier (formic acid) is used prior to the use of this varnish, which interacts with the saliva and enables the interactions of calcium and fluoride [4, 36]. Thus, a stronger, more effective reaction with the enamel surface is expected in use of fTCP, which decreases the porosity rate. Mathias et al. [37] indicated that micro-abrasion plus CPP-ACP significantly decreased the surface roughness. In our study, the use of CPP-ACP decreased the surface roughness but the effect of fTCP was more significant. Agrawal et al. [38] revealed that CPP-ACP, APF and iron supplementation decreased the surface roughness of lesions caused by soft drinks, but this effect was greater with the application of CPP-ACP. The results of Agrawal et al. were not in agreement with ours since they showed that CPP-ACP had greater efficacy for reduction of surface roughness [38]. This difference in the results may be due to the use of different substrates (primary teeth in our study and permanent teeth in the study by Agrawal et al.), difference in the concentration of compounds, difference in the method of application and the duration of use of products, method of assessment of the results, and the depth and pattern of enamel demineralization.

On the other hand, evidence shows that laser irradiation can change the chemical composition of enamel and increase its resistance to acid dissolution and caries [10, 11]. In addition to chemical and structural changes [12], laser irradiation can increase the uptake of fluoride, calcium and phosphate ions [19–22]. This can enhance remineralization and decrease caries. Most previous studies have evaluated the CO_2_ laser [11–18], and studies on diode lasers are limited [19, 20]. The selection of these two lasers in the current study was because they enhance remineralization [39], decrease enamel permeability to chemicals, cause the fusion and recrystallization of enamel crystals, decrease carbonate content (which have a weak bond to apatite crystals and are soluble) and cause organic matrix degradation and obstruction of inter- and intra-prismatic spaces [11–20].

Two theories have been suggested for the mechanism of action of lasers. The first theory explains the increased accumulation of calcium, phosphate and fluoride ions in the cracks and porosities created by lasers. The second theory is based on the penetration of fluoride into the hydroxyapatite and fluorapatite crystals as the result of heat generated by lasers. In the first theory, a weak bond is formed between the tooth structure and ions, and in the second theory, the bond between ions and teeth is strong [40]. On the other hand, laser irradiation causes the obstruction of dentinal tubules by melting them. Additionally, heat generation at the site enables the release of water, organic compounds and carbonate ions from the hydroxyapatite structure, and thus, rate of mineralization increases [41].

The current study showed no significant difference in microhardness after remineralization among the laser groups. However, the microhardness of the diode laser group was slightly higher than that of the CO_2_ laser group. No significant difference existed in percentage of remineralization among laser groups. A small positive synergistic interaction was observed in fTCP + diode laser group. However, it was not statistically significant (*P* > 0.05). Santaella et al. [42] found that the efficacy of materials containing fluoride, calcium and phosphate was higher than the efficacy of diode laser, and diode laser alone was not effective for prevention of lesions. Their results regarding the higher efficacy of fluoride, calcium and phosphate ions were in agreement with ours, but their findings regarding the inefficacy of the diode laser alone was not in line with our results. Kato et al. [43] stated that diode laser alone could not decrease enamel solubility. They used a 960-nm diode laser. The difference between their results and ours may be due to different laser parameters, since we used a 980-nm laser with 7 W power. The laser parameters determine its penetration depth and temperature increase, which determine chemical (low temperature) or morphological (high temperature) changes in dental substrate and adverse effects on the pulp [44]. Laser parameters used in the current study were adopted from a previous study [44]. Vitale et al. [45] and González-Rodríguez et al. [20] showed that diode laser irradiation increases the uptake of ions, especially fluoride, by the enamel structure. The reason was explained to be the thermal changes caused by the laser, increasing the hardness of the tooth surface and subsequently the retention and penetration of fluoride into the surface. Their results supported our findings.

Our results revealed that the porosity rate slightly decreased after remineralization, and the greatest reduction was noted in the CO_2_ laser group. No synergistic effect was noted between materials and lasers. Hossain et al. [46] suggested that the combination of the CO_2_ laser and compounds containing sodium fluoride is more effective for remineralization than CO_2_ laser irradiation alone. Tepper et al. [47] used a CO_2_ laser with 2 W power and 10.6 μm wavelength for 10 s simultaneously with a varnish containing calcium, phosphate and fluoride and showed that this combination increased the acid resistance of enamel samples. We used a 10.6-μm wavelength with 2 W power for 15 s. Rodrigues et al. [13] indicated that CO_2_ laser increased the enamel resistance to demineralization, and this effect was improved when combined with fluoride ions. They reported that the laser was more effective than fluoride for the prevention of caries. The reason may be the fact that they used fluoridated toothpaste as the source of fluoride, which has a lower concentration of fluoride and is less effective than varnishes. Apel et al. [48] stated that the laser-fluoride and laser groups yielded higher enamel resistance to acid corrosion compared to the fluoride group. Rechmann et al. [49] indicated that the CO_2_ laser, with or without the presence of materials containing calcium, phosphate and fluoride ions, decreased the prevalence of caries with no change in the enamel surface. Rocha et al. [15] demonstrated that stannous fluoride alone had no effect on enamel resistance, but the CO_2_ laser alone and in combination with fluoride was significantly effective for this purpose.

In the current study, we tried our best to eliminate the confounding factors. The collected teeth belonged to patients in the same age range, and the remineralizing solution was used with a controlled pH to simulate the oral environment. All procedures were performed by one operator. However, this study had an in vitro design, and thus, the generalization of results to the clinical setting must be done with caution.

One potential limitation of our study was the relatively small sample size, which might have influenced the results. Future studies with larger sample size are recommended to assess the efficacy of different laser types to prevent caries and enhance remineralization. Additionally, the effect of heat generation by lasers on the tooth surface and dental pulp should be studied to find the safest and most effective wavelength and power of lasers for clinical use.

## Conclusion

The highest microhardness was achieved after remineralization with CPP-ACP. The efficacy of diode and CO_2_ lasers was the same. No synergistic effect was found between materials and lasers. The AFM images showed that fTCP reduced the surface roughness more than other materials, and CO_2_ laser decreased the surface roughness more than diode laser. However, further studies are required to increase the generalizability of the current results.

## Data Availability

All materials described in this manuscript including all relevant raw data, will be freely available to any scientist wishing to use them for non-commercial purposes, without breaching participant confidentiality. The data of this research is available from Ehsan Bahrampour (corresponding author).

## Abbreviations

AFM: Atomic force microscopy
CPP-ACP: Casein phosphopeptide amorphous calcium phosphate
fTCP: Functionalized tricalcium phosphate
REMH: Recovery of enamel microhardness
TCP: Tricalcium phosphate
VHN: Vickers hardness number
